# Visceral fat reduction is positively associated with blood pressure reduction in overweight or obese males but not females: an observational study

**DOI:** 10.1186/s12986-019-0369-0

**Published:** 2019-07-10

**Authors:** Xiaohui Guo, Yifan Xu, Hairong He, Hao Cai, Jianfen Zhang, Yibin Li, Xinyu Yan, Man Zhang, Na Zhang, Rolando L. Maddela, Guansheng Ma

**Affiliations:** 1 Department of Nutrition and Food Hygiene, School of Public Health, Peking University, 38 Xue Yuan Road, Haidian District, Beijing, 100191 China; 2 Beijing Key Laboratory of Toxicological Research and Risk Assessment for Food Safety, School of Public Health, Peking University, 38 Xue Yuan Road, Haidian District, Beijing, 100191 China; 3 USANA Health Sciences, Inc., 3838 W Parkway Boulevard, West Valley City, UT 84120 USA

**Keywords:** Visceral fat, Blood pressure, Overweight, Obesity, Meal replacement, Gender

## Abstract

**Background:**

Visceral adiposity has been reported to play a key role in hypertension compared with other measurements of regional or general obesity. The aim of current study was to evaluate the relationship between visceral fat reduction and changes in blood pressure in a group of overweight or obese Chinese individuals.

**Methods:**

An observational study was conducted with 168 participants (ChiCTR-OOC-17012000). Body composition, blood parameters and blood pressure were assessed at the beginning and end of the intervention. Males and females were categorized separately into quartiles according to changes in visceral fat during the intervention. Multiple linear regression models were used to assess the associations of changes in systolic and diastolic blood pressure with changes of visceral fat area, adjusted for potential confounders.

**Results:**

Changes in visceral fat was significantly associated with systolic and diastolic blood pressure in men for systolic (β = 0.234, 95% CI: 0.103, 0.365; *p* = 0.001) and diastolic blood pressure (β = 0.237; 95% CI: 0.127, 0.346; *p* <0.001), but not in women after adjustment for the same potential confounders for systolic blood (β = − 0.003, 95% CI: − 0.260, 0.255; *p* = 0.984) and diastolic blood pressure (β = 0.101, 95% CI: − 0.072, 0.273; *p* = 0.249).

**Conclusions:**

A positive association was observed between reduction in visceral fat and improvements in both systolic blood and diastolic blood pressures in males but not females in a 12-week meal replacement intervention.

**Trial registration:**

The Ethics Committee of Peking University Health Science Center approved the study protocol on 6 July 2017. The authors confirm that all ongoing and related trials for this intervention were carried out following the rules of the Declaration of Helsinki of 1975 and registered (ChiCTR-OOC-17012000). http://www.chictr.org.cn/showprojen.aspx?proj=20426

**Electronic supplementary material:**

The online version of this article (10.1186/s12986-019-0369-0) contains supplementary material, which is available to authorized users.

## Background

Cardiovascular disease is the leading cause of morbidity and mortality worldwide and hypertension is an independent cardiovascular risk factor [1]. The incidence of hypertension is predicted to increase by 60% over the next 30 years [2]. According to the Report on Chinese Residents’ Chronic Diseases and Nutrition, the overall occurrence of hypertension in Chinese adults was 25.2% in 2012 and rising drastically due to the rapid urbanization of China [3–6], accompanied by increases in chronic non-communicable diseases, such as kidney cancer, ischemic heart disease, and cerebrovascular disease [7, 8]. However, more than 125 million people with hypertension (11.9% of the Chinese adult population) are unaware of their condition thus only 15.3% of people (37 million) with hypertension are controlled [9]. The control of hypertension is critically important because it is one of the leading preventable risk factors for premature death and disability [10].

Overweight and obesity are associated with the emergence of hypertension and it has been shown that weight loss leads to blood pressure reduction [11, 12]. Obesity can be measured by the body mass index (BMI), however, this is not able to distinguish between lean and fat mass and does not provide indication of body fat distribution [13]. Compared to BMI, abdominal obesity measures such as waist circumference or visceral fat, are better at predicting cardiovascular disease and its risk factors [14]. However, waist circumference measurement is not as precise as visceral fat because it is a function of both the subcutaneous adipose tissue and visceral adipose tissue compartments [15].

Visceral fat has been reported to be associated with blood pressure [15–17]. Several cross sectional studies in US populations have found positive associations between visceral fat and BP in Caucasian, African American and Japanese American subjects [18, 19]. A Framingham cohort found the link between visceral fat deposition and blood pressure, as well as the prevalence of hypertension [15]. However, there are limited studies investigating the association between BP and visceral adiposity in Chinese populations [20]. A cross-sectional study conducted in China found that visceral body fat is strongly associated with higher risk of hypertension and prehypertension [21]. Notwithstanding the presence of observational studies connecting visceral fat deposition to BP, there are only a few studies that address the effects of visceral fat reduction on the changes of blood pressure in overweight and obese subjects.

In relation, the aim of the current study was to evaluate the relationship between visceral fat reduction and BP changes among a group of overweight or obese Chinese subjects in a 12-week meal replacement weight loss intervention.

## Methods

### Ethics statement

The study was approved by the Ethics Committee of Peking University Health Science Center on July 6, 2017. All of the subjects were informed of procedures and signed a consent form prior to commencement. The authors confirm that all ongoing and related trials for this intervention were carried out following the rules of the Declaration of Helsinki of 1975 and registered with the Chinese Clinical Trial Registry (ChiCTR-OOC-17012000).

### Study volunteers

Free living participants were recruited through advertisements in and around the Beijing area. Briefly, potential participants, aged 18–55 years, were required to complete a basic screening questionnaire to assess eligibility. Individuals with a BMI > 24 kg/m^2^ who: (1) were not actively trying to lose weight, (2) did not have allergies to any of the known food ingredients, and (3) were not pregnant or currently breast feeding were included in the study. Subjects were excluded from the study if they had: (1) physician-diagnosed cognitive impairment, schizophrenia, or depression by a physician, (2) heavy alcohol consumption, defined as 61 g alcohol drinks per day for male and 41 g alcohol drinks per day for females [22], which were collected by screening before randomization, (3) pacemaker or other internal electronic medical device, or (4) irregular diet or work schedules such as night shift.

### Study design

This is an observational study that looked at data from a randomized controlled clinical trial that was conducted among free-living 168 overweight or obese subjects over a period of 3 months. The details of the original study have been published [23]. Briefly, the participants in the study were randomly assigned into 2 groups, the meal replacement group (intervention group) and the routine diet group (control group). The subjects in the intervention group were required to consume a meal replacement, which contains 22.6 g protein, 11.1 g fat, 39.3 g carbohydrate, 20.9 g dietary fiber and 388 kcal in total energy at dinner time while the participants in the control group were advised to continue a routine diet as before. The intervention group participants were provided meals to last until the next visit at no charge. Study participants were scheduled to come back every 4 weeks for assessments. If a subject was unable or unwilling to regularly attend the scheduled appointments, that participant was considered a drop-out.

### Outcome measures

Demographic information including lifestyle, health condition, education, history of illnesses, and medication use were collected by a brief self-administered 17-item general questionnaire. Dietary habits were assessed through a self-administered 77-item Food Frequency Questionnaire (FFQ) at the beginning and last visit. Physical activity was evaluated by a self-administered 24-item questionnaire.

Height was measured using a standard wall-mounted stadiometer to the nearest 0.5 cm. BMI was calculated as weight in kilograms divided by height in meters squared. Overweight was defined as 24 ≥ BMI < 28 kg/m^2^ and obesity was defined as BMI ≥ 28 kg/m^2^ according to the current definitions for Chinese adults [24]. Body composition was measured by using multi-frequency bioelectrical impedance analysis with 8-point tactile electrodes (InBody 720; Biospace, Seoul, Korea) [23]. Bioelectrical impedance was measured within 1–2 min with the subject standing in her/his bare feet and grasping the hand electrodes with arms in the vertical position. Body composition parameters included waist to hip ratio (WHtR), fat-free mass (FFM), body fat mass (BFM), visceral fat area (VFA), and body fat percent (BFP).

BP measurements were determined by a validated semiautomatic sphygmomanometer (Omron HEM-705CP) by trained nurses. Two measurements were taken at 5-min intervals with participants in a seated position. Data were reported as an average of 2 measurements [25]. Hypertension was defined as systolic blood pressure (SBP) higher than 140 mmHg or diastolic blood pressure (DBP) higher than 90 mmHg [24].

### Statistical analysis

To ensure that the data met assumptions of parametric tests, these were assessed for outliers, homoscedasticity, and normality using Kolmogorov and Levene tests. One- way ANOVA was employed for multiple comparisons. Changes of VFA during the study were calculated by VFA at last visit minus VFA at baseline.

Multiple linear regression models were used to assess the association of changes in SBP and DBP, with changes in visceral fat area during the intervention period, adjusted for potential confounders (Model 1: unadjusted; Model 2: adjusted for age, intervention groups, body weight, BMI, respectively; Model 3 adjusted as in Model 2 plus smoking status, education level, marital status, diabetes, dyslipidemia, and medication use at baseline: antihypertensive drugs, oral hypoglycemic drugs; Model 4 was adjusted as in Model 3 energy intake and physical activity at baseline. Covariates were selected according to previous studies which identified potential modifiers of body weight and BP [21, 26, 27]. The factor model that fit the data was evaluated by using the Akaike Information Criterion (AIC) [28].

All analyses were performed using SPSS software V24.0 (SPSS Inc., Chicago, IL, USA,). Results were expressed as mean ± SD for continuous variables or percentages for categorical variables. All statistical tests were two-tailed, and the threshold for significance level was *p* < 0.05.

**Fig. 1:**
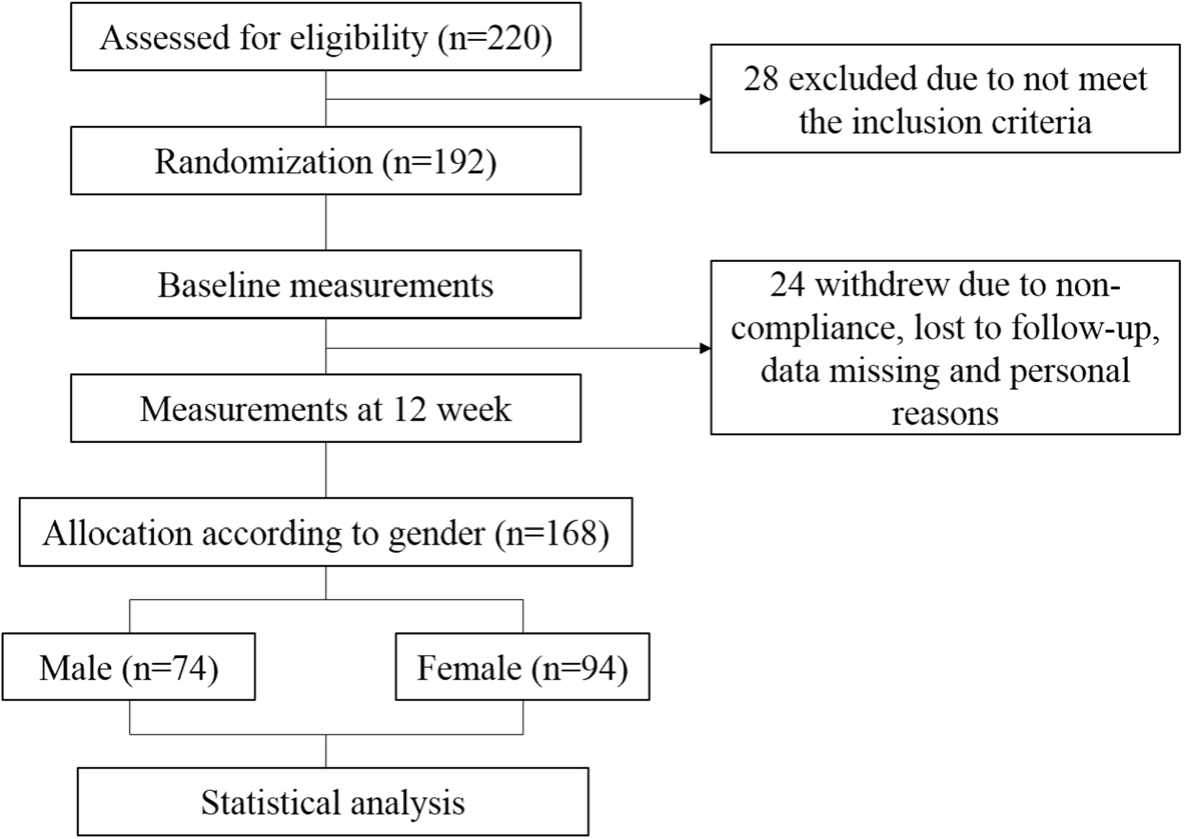
Flowchart of participants

## Results

As shown in the Fig. 1, A total of 220 subjects were screened from Beijing and the surrounding areas. Twenty-eight individuals were excluded because they did not meet the inclusion criteria. During the study, 24 withdrew for various reasons: not able to meet the scheduled appointment, non-compliance and personal reasons, hence a total of 168 participants were finally included.

The baseline characteristics of participants are shown in Table 1. Seventy-four male individuals and ninety-four female individuals were included in the study. There were differences between males and females in body weight, BMI, systolic and diastolic blood pressure, prevalence of hypertension and dyslipidemia, smoking status, use of antihypertensive drugs and visceral fat area at baseline (*p* < 0.05).

**Table 1:** Baseline characteristics of participants

	Male	Female	p*
No. of subjects	74	94	
Age (y), mean (SD)	38.4 ± 6.6	38.1 ± 7.9	0.760
Weight (kg), mean (SD)	89.6 ± 11.2	73.4 ± 8.0	< 0.001
BMI (kg/m^2^), mean (SD)	30.0 ± 2.8	28.8 ± 3.0	0.011
Systolic BP (mm Hg), mean (SD)	134.1 ± 15.2	120.6 ± 16.3	< 0.001
Diastolic BP (mm Hg), mean (SD)	88.7 ± 12.1	78.4 ± 12.2	< 0.001
Hypertension, *n* (%)	19 (20.4)	42 (53.2)	< 0.001
Diabetes, *n* (%)	3 (3.3)	5 (6.5)	0.332
Dyslipidemia, *n* (%)	18 (19.4)	26 (32.5)	0.048
Smoking status, *n* (%)			0.042
Smoker	13 (16.3)	6 (6.4)	
None-smoker	67 (83.8)	88 (93.6)	
Medication, *n* (%)			
Aspirin	0 (0)	0 (0)	–
Antihypertensive drugs	10 (12.5)	3 (3.2)	0.021
Hypolipidemic drugs	5 (6.3)	2 (2.1)	0.168
Insulin	0 (0)	0 (0)	–
Oral hypoglycemic drugs	2 (2.5)	2 (2.1)	0.870
Vitamin	8 (10.0)	9 (9.6)	0.925
Minerals	6 (7.5)	7 (7.4)	0.989
Education level, (%)			0.478
University	63 (78.8)	78 (83.0)	
High school	17 (21.3)	16 (17.0)	
Primary school	0 (0)	0 (0)	
Marital status, (%)			0.352
Single	6 (7.5)	11 (11.7)	
Married	74 (92.5)	83 (88.3)	
Widowed	0 (0)	0 (0)	
VFA (cm^2^)	126.0 ± 34.3	145.0 ± 30.9	< 0.001

*BMI* body mass index, *BP* blood pressure, *VFA* visceral fat area. Data are given as means (SD) for continuous variables and percentages for categorical variables; *p* < 0.05 indicates statistical significance. *p-values calculated by analysis of variance or χ2 tests

Body composition parameters (BW, BMI, WC, WHtR, FFM, BFM, BFP, VFA) and blood pressures (SBP, DBP), stratified by genders are shown in Table 2. Comparing males with females, significant differences (*p* < 0.05) were found in BW, BMI, WC, WHtR, BFP, SBP and DBP before and after the study. Significant changes (p < 0.05) were only found in WHtR, and systolic and diastolic blood pressures.

**Table 2:** Characteristic of body composition parameters and blood pressure before and after the study among males and females^a^

		Male(*n* = 74)	Female(*n* = 94)	*p* ^b^
BW (kg)	Baseline	89.6 ± 11.2	73.4 ± 8.0	< 0.001
	Changes	−2.0 ± 3.7	−1.6 ± 2.6	0.486
	Final	87.5 ± 11.1	71.7 ± 8.7	< 0.001
BMI (kg/m^2^)	Baseline	30.0 ± 2.8	28.8 ± 3.0	0.011
	Changes	−0.6 ± 1.2	−0.6 ± 1.0	0.994
	Final	29.3±3.0	28.2 ± 3.2	0.02
WC (cm)	Baseline	102.5 ± 7.2	93.1 ± 7.6	< 0.001
	Changes	−2.2 ± 4.5	−1.4 ± 4.3	0.219
	Final	100.3 ± 7.6	91.7 ± 8.1	< 0.001
WHtR	Baseline	0.96 ± 0.06	0.93 ± 0.05	< 0.001
	Changes	0.02 ± 0.05	0.03 ± 0.03	0.017
	Final	0.98 ± 0.08	0.96 ± 0.06	0.037
FFM (kg)	Baseline	61.4 ± 6.6	44.2 ± 4.2	< 0.001
	Changes	−0.5 ± 1.5	−0.6 ± 1.1	0.779
	Final	60.9 ± 6.6	43.6 ± 4.4	< 0.001
BFM (kg)	Baseline	28.2 ± 6.7	29.7 ± 5.7	0.283
	Changes	−0.6 ± 3.4	0.1 ± 2.1	0.137
	Final	27.5 ± 7.6	29.3 ± 6.2	0.101
BFP (%)	Baseline	31.1 ± 4.7	39.6 ± 4.3	< 0.001
	Changes	−0.4 ± 2.9	0.3 ± 1.9	0.92
	Final	30.7 ± 5.9	39.8 ± 4.7	< 0.001
VFA (cm^2^)	Baseline	126.0 ± 34.3	145.0 ± 30.9	< 0.001
	Changes	−0.4 ± 20.2	4.1 ± 12.9	0.082
	Final	125.1 ± 42.0	149.1 ± 34.9	< 0.001
SBP (mmHg)	Baseline	134.1 ± 15.2	120.6 ± 16.3	< 0.001
	Changes	−3.8 ± 9.8	1.7 ± 13.9	0.005
	Final	129.9 ± 14.0	122.3 ± 15.8	0.001
DBP (mmHg)	Baseline	88.7 ± 12.1	78.4 ± 12.2	< 0.001
	Changes	−4.7 ± 8.7	−0.9 ± 9.4	0.008
	Final	83.7 ± 9.6	77.5 ± 11.4	< 0.001

^a^Data are given as means (SD); *p* < 0.05 indicates statistical significance. ^b^Data were analyzed by one-way ANOVA;

*BW* body weight, *BMI* body mass index, *WC* waist circumference, *WHtR* waist to hip ratio, *FFM* fat free mass, *BFM* body fat mass, *BPF* body fat percent, *SBP* systolic blood pressure, *DBP* diastolic blood pressure

Linear regression analyses were conducted to assess the relationship between changes in VFA and changes in SBP and DBP during the study duration (Table 3). Significant positive associations were found in males for systolic (β = 234, 95% CI: 0.103, 0.365; *p* = 0.001) and diastolic blood pressure (β = 0.237; 95% CI: 0.127, 0.346; *p* <0.001), but not in females after adjustment for the same potential confounders for systolic blood (β = − 0.003, 95% CI: − 0.260, 0.255; *p* = 0.984) and diastolic blood pressure (β = 0.101, 95% CI: − 0.072, 0.273; *p* = 0.249).

**Table 3:** Association between changes of visceral fat and blood pressure

			β	SE	Sig.	95% CI		AIC
Changes in SBP	Male	Model 1	0.128	0.058	0.032	0.012	0.245	350.888
		Model 2	0.166	0.060	0.008	0.045	0.286	349.638
		Model 3	0.197	0.060	0.002	0.076	0.319	341.329
		Model 4	0.234	0.065	0.001	0.103	0.365	337.184
	Female	Model 1	0.017	0.114	0.882	−0.209	0.243	496.459
		Model 2	0.003	0.116	0.981	−0.229	0.234	499.331
		Model 3	−0.032	0.124	0.799	−0.279	0.216	503.544
		Model 4	−0.003	0.129	0.984	−0.260	0.255	503.959
Changes in DBP	Male	Model 1	0.132	0.052	0.013	0.029	0.236	332.028
		Model 2	0.168	0.052	0.002	0.063	0.272	326.703
		Model 3	0.186	0.055	0.001	0.077	0.296	325.275
		Model 4	0.237	0.055	<0.001	0.127	0.346	309.072
	Female	Model 1	0.052	0.077	0.495	−0.100	0.205	422.278
		Model 2	0.060	0.079	0.451	−0.097	0.217	426.542
		Model 3	0.086	0.082	0.300	−0.078	0.250	425.930
		Model 4	0.101	0.087	0.249	−0.072	0.273	428.551

*SBP* systolic blood pressure, *DBP* diastolic blood pressure

*SE* standard error, *CI* confidence interval, two-sided test of significance, *AIC* Akaike’s Information Criterion

Model 1: unadjusted;

Model 2: adjusted for age, intervention groups, body weight, BMI;

Model 3 adjusted as in Model 2 plus smoking status, education level, marital status, diabetes, dyslipidemia, and medication use at baseline: antihypertensive drugs, oral hypoglycemic drugs;

Model 4 was adjusted as in Model 3 plus energy intake and physical activity at baseline

## Discussion

A 12-week meal replacement intervention conducted on a group of overweight or obese Chinese subjects in and around the Beijing area demonstrated a significant reduction in visceral fat in males but not in females. After regression analysis, positive associations were found between reduction in visceral fat and improvement in SBP and DBP in males but not females. This finding highlights the importance of visceral fat reduction in interventions for hypertension and suggests further that weight loss and intervention programs for hypertension should be designed to include assessment of variability in response due to gender.

In comparison to other measurements of obesity such as BMI, waist circumference and subcutaneous adipose tissue, the available studies report that the measurement of visceral fat shows more association with its increase with higher prevalence of hypertension [12, 18, 20, 29, 30]. With linear regression analysis, we found significant relationship of VFA with SBP in males at baseline, and also of the reduction in VFA with improvement of SBP and DBP in males after the intervention, which is consistent with findings of with previous studies. Ideally, the assessment of visceral fat requires imaging with radiographic techniques such as computed tomography (CT), magnetic resonance imaging (MRI), and dual-energy X-ray absorptiometry (DEXA). However, these techniques are not readily available and are costly. Bioelectrical impedance analyzer (BIA) is a good alternative to estimate visceral fat [31] because it is more convenient and less expensive. In the current study, a validated, multi-frequency BIA (Inbody 720®) was used to assess the body composition, which presented highly reliable results to support our findings.

Previous studies with cross sectional approaches have examined the association between visceral fat and blood pressure but limited investigations have shown the relationship between the reduction in visceral fat and improvements in blood pressure during short interventions similar to this study. Positive associations between visceral fat and hypertension were found in other populations such as Caucasian, African American, and Japanese Americans, as well as French-Canadians [18, 19, 32]. For the Chinese population, a cross-sectional study found positive associations between visceral fat and prevalence of prehypertension and hypertension [21]. Studies elsewhere show the greater beneficial effects of visceral fat reduction compared to subcutaneous fat reduction. A 12-week longitudinal intervention study conducted among visceral obese and subcutaneous obese subjects, found significant reduction in both SBP (− 8.2 mmHg) and DBP (− 4.6 mmHg) after a 37.3 cm^2^ visceral fat decrement [33]. Another study mentions that both volume and quality of visceral and subcutaneous fat to metabolic risk including hypertension [34]. Other findings may not show the association of visceral fat and hypertension like in a 3-month weight-loss intervention study showed significant reduction in lean leg mass, instead of visceral fat, was positively associated with systolic blood pressure [35]. Another study concluded that waist circumference had an advantage over visceral fat thickness as an obesity index to identify components of metabolic syndrome that included hypertension [36]. In spite of some findings of negative associations between visceral fat and metabolic risks, there are still more published data about incident associations of metabolic risks that include hyperetnsion and visceral fat. Our findings contribute to the growing research related to the association between visceral fat and risk of hypertension [37].

After multivariate adjustment, the association between visceral fat reduction and improvement in SBP and DBP was increased in male subjects, indicating that relationship between visceral fat reduction and BP decrement in men was independent of body weight, BMI and other potential confounders. However, such association was not found in females even after multiple adjustments, which shows that gender difference may exist when exploring the effects of visceral fat reduction on BP changes.

There are several plausible mechanisms that may explain the relationship between reduction in visceral fat and improvement in BP. First, previous studies have demonstrated that visceral fat is associated with secretion of adipocytokines, which contribute to the development and progression of BP elevation [15, 38, 39]. Second, visceral fat contributes free-fatty acids through the portal vein, which may lead to insulin resistance which was shown to be associated with the prevalence and incidence of hypertension [39–41]. Third, cardiac sympathetic activity was observed to be higher in visceral obesity than in subcutaneous obesity, which suggests possible link between blood pressure and visceral fat [42]. Fourth, visceral fat may be associated with activation of the renin-angiotensin-aldosterone system which has been implicated in obesity-associated hypertension [43].

The differences observed between genders in the current study might be explained by multiple possibilities. First, visceral adipose tissue is lost preferentially with short term and modest weight loss, but the effect is attenuated with extension of time and greater weight loss [16]. In our study, males tend to have higher weight but less visceral fat than females then after the intervention, the males lost more body weight and visceral fat than females. Therefore, the association between visceral fat reduction and improvement in BP was stronger in males than females. Also, gender differences observed in the study might be attributed to the variability in hormone levels, body fat percent, fat distribution, attitudes and behaviors towards the same dietary intervention program [44–47]. However, we did not assess these potential factors in the current study.

Some limitations of this study should be noted. First, the length of the intervention was only 12 weeks and a longer duration may be more informative and enhance nominally significant associations in light of our modest caloric intervention. Second, the sample size was relatively small thus it was difficult to perform further stratified analysis by quartile. Third, the self-administered food questionnaire and physical activity questionnaire are not able to provide sufficient precise data to further analyze and support the observed differences between genders. Fourth, the study was not designed to identify biochemical biomarkers, therefore we cannot analyze the potential mechanisms underlying changes in BP in response to insulin sensitivity and adipocytokines. Fifth, sodium and potassium intake are associated with studies on hypertension, however, those data were not obtained in the present study.

The strengths of our work mainly include the longitudinal study design that showed the relationship between visceral fat reduction and BP improvements in a weight loss program, thus, expanded upon prior work that has shown associations between visceral fat and incident hypertension. Second, the analysis performed under each gender setting showed different results between genders which suggests that further exploration of potential mechanisms is necessary.

## Conclusions

In summary, our study found a significant correlation between the reduction of visceral fat area and improvements in systolic and diastolic BP in overweight or obese male subjects, confirming the importance of visceral fat reduction in a weight loss program on the relevant health outcome. Our findings indicate that the evaluation of changes in visceral fat may be necessary when designing intervention programs for hypertension. Furthermore, research regarding gender-specific associations between visceral fat and blood pressure changes is warranted.

## Additional file

Additional file 1: Database of participants. (XLSX 88 kb)

## Data Availability

The dataset supporting the conclusions of this article is included within the Additional file 1. Database of participants. Individual deidentified participant data is available, including basic information, body composition and BP parameters. Other documents, such as study protocol, statistical analysis plan is not available.

## Abbreviations

BCM: Body cell mass
BFM: Body fat mass
BMI: Body mass index
DBP: Diastolic blood pressure
FFM: Fat-free mass
SBP: Systolic blood pressure
VFA: Visceral fat area
WHtR: Waist to hip ratio
